# The Inpatient Burden of Pediatric Dermatology in the United States: A National Analysis of the 2022 Kids' Inpatient Database

**DOI:** 10.1111/pde.70151

**Published:** 2026-02-13

**Authors:** Shiv Patel, Aayush Shah, Vibhusha Kolli, Peter A. Lio

**Affiliations:** ^1^ Feinberg School of Medicine Northwestern University Chicago Illinois USA; ^2^ University of Florida Gainesville Florida USA; ^3^ School of Medicine and Public Health University of Wisconsin Madison Wisconsin USA

## Abstract

**Background:**

Dermatologic conditions are a significant reason for pediatric hospitalizations in the United States. A prior analysis of the 2012 Kids' Inpatient Database (KID) established a national benchmark for this burden, but changes in medical coding, healthcare delivery, and demographics necessitate an updated assessment. This study aimed to quantify the current inpatient burden of pediatric dermatology and to identify associated demographic risk factors, financial costs, and mortality using a recent, nationally representative database.

**Methods:**

A cross‐sectional study was conducted using the 2022 KID. Pediatric hospitalizations with a primary dermatology diagnosis (ICD‐10‐CM) were identified. Survey weights were used to generate national estimates of admission counts, costs, and mortality. Multivariable logistic regression was used to identify independent risk factors for a primary dermatology hospitalization.

**Results:**

In 2022, there were an estimated 29,766 pediatric dermatology hospitalizations, representing 2.3% of all pediatric admissions and a decline from 4.2% in 2012. These admissions generated a total national cost of $449.3 million. After adjusting for covariates, factors associated with higher odds of a dermatology admission included age 2–5 years (OR: 1.35; 95% CI: 1.21–1.52), Asian/Pacific Islander race (OR: 1.49; 95% CI: 1.32–1.69), Native American race (OR: 1.17; 95% CI: 1.00–1.36), Hispanic ethnicity (OR: 1.07; 95% CI: 1.01–1.14), lowest income quartile (OR: 1.06; 95% CI: 1.01–1.12), and Medicaid coverage (OR: 1.09; 95% CI: 1.04–1.14). Female sex was associated with lower odds (OR: 0.95; 95% CI: 0.92–0.98). In‐hospital mortality was 0.1%.

**Conclusion:**

The national burden of inpatient pediatric dermatology has decreased over the past decade. However, significant disparities related to socioeconomic status and race/ethnicity persist and have evolved. These findings underscore the continued need for interventions aimed at improving access to outpatient dermatologic care for underserved pediatric populations.

## 1 Introduction

Inpatient care represents a substantial proportion of U.S. healthcare expenditures, and dermatologic conditions continue to account for a meaningful share of pediatric hospitalizations [1, 2]. Although most pediatric skin diseases are managed in the outpatient setting, certain infections, congenital disorders, and inflammatory conditions require inpatient admission and generate significant costs [3].

National data describing the burden of pediatric dermatology hospitalizations have historically been limited. Earlier work, including the analysis of the 2012 Kids' Inpatient Database (KID) by Arnold et al., reported more than 74,000 dermatology‐related admissions, comprising 4.2% of all pediatric hospitalizations and nearly $380 million in associated healthcare costs [4]. That study also identified disparities in risk, with higher admission rates among children from low‐income communities, those residing in the South, and patients with Medicaid or no insurance.

Since 2012, the healthcare landscape has changed considerably. In October 2015, the United States implemented the transition from ICD‐9‐CM to ICD‐10‐CM/PCS coding, expanding diagnostic detail from roughly 17,000 to more than 150,000 unique codes [5, 6]. This transition has improved the specificity of hospital discharge data and allows for more precise categorization of dermatologic conditions. In addition, evolving insurance coverage patterns and heightened attention to healthcare disparities may have altered the epidemiology of pediatric dermatology admissions [7, 8, 9].

The objective of this study was to replicate and update the findings of Arnold et al. using the 2022 KID, the most recent nationally representative pediatric inpatient dataset. The study aimed to quantify the current burden of pediatric dermatology hospitalizations, identify demographic and socioeconomic risk factors, and assess the financial costs and mortality associated with these admissions.

## 2 Materials and Methods

A cross‐sectional study of pediatric dermatology hospitalizations in the United States was performed using the 2022 Kids' Inpatient Database (KID), a nationally representative sample maintained by the Healthcare Cost and Utilization Project (HCUP). This study was conducted using de‐identified data from KID. As such, it was deemed exempt from institutional review board oversight and informed consent requirements. All authors with access to the data complied with the HCUP Data Use Agreement. There was no authorship overlap between the present study and the 2012 analysis by Arnold et al.

The 2022 database includes discharges from 46 states and the District of Columbia, representing a slight expansion in coverage from the 2012 KID, which included data from 44 states and allows for weighted estimates for all pediatric hospitalizations nationwide. Consistent with prior work, admissions for pregnancy, childbirth, and routine newborn care were excluded. Dermatology hospitalization was defined as one with an ICD‐10‐CM dermatologic code recorded as the primary diagnosis (see Table S1 for category code lists). Admissions in which skin disease appeared only as a secondary diagnosis were not counted, such as a chemotherapy admission complicated by a cutaneous adverse event or sepsis with an associated exanthem. For conditions such as reactive infectious mucocutaneous eruption (RIME) that lack a unique ICD‐10‐CM code and are typically coded under related entities, cases were counted only when those related codes were recorded as the primary diagnosis.

Cases were classified into 21 disease categories to maintain direct comparability with the methodology of the foundational 2012 analysis by Arnold et al. This classification scheme was adapted from the 24 categories defined in the American Academy of Dermatology's 2016 Burden of Skin Disease report [10]. The corresponding ICD‐10‐CM codes for each category were identified using the official General Equivalence Mappings (GEMs) provided by the Centers for Medicare & Medicaid Services (CMS) and subsequently reviewed for clinical accuracy. Consistent with the methodology of the prior analysis, conditions of the external ear, genitalia, eyelids, and lips were excluded.

Demographic variables included age, sex, race and ethnicity, insurance status, income quartile of the patient's zip code, and geographic region. Hospital characteristics included ownership, bed size, and teaching status. Costs were derived from hospital charges using HCUP‐provided cost‐to‐charge ratios. The primary outcomes were the national burden of pediatric dermatology hospitalizations, demographic and socioeconomic risk factors, and financial costs; in‐hospital mortality was examined as a secondary outcome. Analyses were conducted using survey weights to generate national estimates. Differences between groups were tested with Rao–Scott adjusted chi‐square tests for categorical variables and design‐based Kruskal–Wallis tests for continuous variables. Multivariable logistic regression was used to identify independent predictors of dermatology hospitalization, with results reported as odds ratios and 95% confidence intervals. The model included all patient and hospital characteristics listed in Table 1 as covariates.

**TABLE 1:** Weighted characteristics of pediatric hospitalizations, KID 2022.

Characteristic	Overall (*N* = 1,312,198)	Non‐dermatology (*N* = 1,282,432)	Dermatology (*N* = 29,766)	*p*
Age group				< 0.001
< 29	29,923 (2.3%)	29,324 (2.3%)	599 (2.0%)	
29 days–1 years	216,754 (17%)	212,767 (17%)	3986 (13%)	
2–5 years	389,253 (30%)	378,724 (30%)	10,529 (35%)	
6–9 years	164,680 (13%)	159,968 (12%)	4712 (16%)	
10–13 years	200,586 (15%)	196,409 (15%)	4177 (14%)	
14–17 years	311,003 (24%)	305,240 (24%)	5763 (19%)	
Sex				< 0.001
Male	683,685 (52%)	667,664 (52%)	16,021 (54%)	
Female	628,513 (48%)	614,769 (48%)	13,745 (46%)	
Race/ethnicity				< 0.001
White	628,461 (48%)	615,198 (48%)	13,263 (45%)	
Black	229,725 (18%)	224,562 (18%)	5164 (17%)	
Hispanic	314,015 (24%)	306,449 (24%)	7565 (25%)	
Asian/Pacific Islander	47,825 (3.6%)	46,312 (3.6%)	1513 (5.1%)	
Native American	13,684 (1.0%)	13,334 (1.0%)	350 (1.2%)	
Other	78,488 (6.0%)	76,577 (6.0%)	1911 (6.4%)	
Income quartile				< 0.001
1 (lowest)	387,549 (30%)	378,276 (29%)	9274 (31%)	
2	325,456 (25%)	317,988 (25%)	7467 (25%)	
3	318,595 (24%)	311,718 (24%)	6877 (23%)	
4 (highest)	280,598 (21%)	274,451 (21%)	6148 (21%)	
Region				0.011
West	289,476 (22%)	282,996 (22%)	6480 (22%)	
Northeast	240,304 (18%)	234,746 (18%)	5558 (19%)	
Midwest	290,456 (22%)	284,545 (22%)	5911 (20%)	
South	491,963 (37%)	480,144 (37%)	11,818 (40%)	
Primary payer				< 0.001
Private	510,122 (39%)	499,303 (39%)	10,820 (36%)	
Medicaid	703,286 (54%)	686,415 (54%)	16,871 (57%)	
Medicare	3026 (0.2%)	2987 (0.2%)	38 (0.1%)	
Self‐pay	33,750 (2.6%)	32,899 (2.6%)	851 (2.9%)	
No charge	1061 (< 0.1%)	1028 (< 0.1%)	33 (0.1%)	
Other	60,953 (4.6%)	59,801 (4.7%)	1153 (3.9%)	
Length of stay (days), median (IQR)	3 (1–5)	3 (1–5)	2 (2–4)	< 0.001
Cost ($), median (IQR)	7931 (4186–16,784)	7965 (4197–16,891)	6728 (3774–12,849)	< 0.001
In‐hospital mortality	6222 (0.5%)	6180 (0.5%)	42 (0.1%)	< 0.001

## 3 Results

In 2022, there were an estimated 29,766 pediatric dermatology hospitalizations in the United States, representing 2.3% of all pediatric admissions and generating a total national cost of $449.3 million (95% CI: $379.7 million–$518.8 million).

Compared with non‐dermatology admissions, dermatology hospitalizations had a higher proportion of children aged 2–5 years (35% vs. 30%) and of Hispanic (25% vs. 24%), Asian/Pacific Islander (5.1% vs. 3.6%), and Native American (1.2% vs. 1.0%) patients; females were slightly less represented (46% vs. 48%). Patients were more often from the lowest income quartile (31% vs. 29%) and insured by Medicaid (57% vs. 54%). The South accounted for 40% of dermatology admissions. Detailed demographic distributions are reported in Table 1. After adjusting for patient and hospital factors (Table 2), hospitalization for skin disease was associated with age 2–5 years (OR: 1.35; 95% CI: 1.21–1.52), Asian/Pacific Islander race (OR: 1.49; 95% CI: 1.32–1.69), Native American race (OR: 1.17; 95% CI: 1.00–1.36), Hispanic ethnicity (OR 1.07; 95% CI: 1.01–1.14), lowest income quartile (OR 1.06; 95% CI: 1.01–1.12), and Medicaid coverage (OR 1.09; 95% CI: 1.04–1.14). Female sex was associated with lower odds (OR: 0.95; 95% CI: 0.92–0.98).

**TABLE 2:** Adjusted odds ratios for a primary dermatology hospitalization, KID 2022.

Characteristic	OR (95% CI)	*p*
Age group		
< 29	Reference	
29 days–1 years	0.91 (0.81–1.01)	0.089
2–5 years	1.35 (1.21–1.52)	< 0.001
6–9 years	1.43 (1.27–1.61)	< 0.001
10–13 years	1.04 (0.92–1.18)	0.5
14–17 years	0.94 (0.83–1.06)	0.3
Sex		
Male	Reference	
Female	0.95 (0.92–0.98)	< 0.001
Race/ethnicity		
White	Reference	
Black	0.99 (0.95–1.04)	0.8
Hispanic	1.07 (1.01–1.14)	0.02
Asian/Pacific Islander	1.49 (1.32–1.69)	< 0.001
Native American	1.17 (1.00–1.36)	0.048
Other	1.12 (1.04–1.19)	0.001
Income quartile		
4 (highest)	Reference	
1 (lowest)	1.06 (1.01–1.12)	0.032
2	1.04 (0.99–1.10)	0.14
3	0.99 (0.94–1.04)	0.7
Region		
West	Reference	
Northeast	1.05 (0.95–1.15)	0.3
Midwest	0.95 (0.85–1.06)	0.4
South	1.08 (1.00–1.18)	0.066
Insurance		
Private	Reference	
Medicaid	1.09 (1.04–1.14)	< 0.001
Medicare	0.57 (0.39–0.84)	0.005
Self‐pay	1.18 (1.06–1.32)	0.003
No charge	1.36 (0.86–2.13)	0.2
Other	0.86 (0.79–0.95)	0.002
Hospital ownership		
Government	Reference	
Private, nonprofit	1.00 (0.91–1.10)	0.9
Private, for‐profit	1.09 (0.92–1.30)	0.3
Hospital bed size		
Small	Reference	
Medium	0.98 (0.87–1.11)	0.8
Large	0.94 (0.84–1.05)	0.3
Hospital location/teaching		
Rural	Reference	
Urban, non‐teaching	0.96 (0.80–1.15)	0.6
Urban, teaching	0.94 (0.83–1.06)	0.3

Median length of stay for dermatology admissions was 2 days (IQR 2–4), compared with 3 days (IQR: 1–5) for non‐dermatology admissions (*p* < 0.001). Median cost per admission was $6728 (IQR: $3774–$12,849), lower than for non‐dermatology admissions ($7965, IQR: $4197–$16,891; *p* < 0.001). In‐hospital mortality for dermatological cases was rare, occurring in 42 cases (0.1%) compared with pediatric in‐hospital deaths from non‐dermatologic conditions (0.5%). These deaths were concentrated in high‐acuity conditions, including cutaneous lymphoma (12 deaths), viral diseases (8 deaths), and congenital skin abnormalities (8 deaths).

As shown in Table 3, bacterial infections and infestations accounted for the largest share of admissions (~19,300 cases), followed by connective tissue disorders (~3900 cases) and viral diseases (~2700 cases). Although less frequent, cutaneous lymphomas (~770 cases) and congenital skin abnormalities (~230 cases) were the most expensive conditions, with mean costs per admission of $100,573 and $45,521, respectively.

**TABLE 3:** Weighted admission counts and costs by dermatology disease category, KID 2022.

Disease category	National admissions (95% CI)	Mean cost per admission (95% CI)	Total national cost (95% CI)
Bacterial infections/infestations	19,326 (17,676–20,976)	$8133 ($7489–$8776)	$157.2 M ($138.1–$176.2 M)
Connective tissue disorders	3875 (3400–4350)	$31,993 ($28,389–$35,596)	$124.0 M ($102.0–$146.0 M)
Viral diseases	2721 (2419–3023)	$10,963 ($9422–$12,503)	$29.8 M ($24.4–$35.3 M)
Cutaneous lymphoma	766 (642–891)	$100,573 ($85,482–$115,664)	$77.0 M ($57.9–$96.1 M)
Noncancerous skin growths	647 (545–749)	$23,592 ($17,648–$29,536)	$15.3 M ($10.8–$19.8 M)
Other eczema/contact dermatitis	443 (377–509)	$6141 ($5323–$6960)	$2.7 M ($2.2–$3.3 M)
Fungal infections	346 (291–401)	$33,262 ($20,476–$46,048)	$11.5 M ($6.4–$16.6 M)
Urticaria	289 (239–339)	$5786 ($4058–$7515)	$1.7 M ($1.1–$2.2 M)
Ulcers	279 (224–334)	$36,907 ($28,504–$45,310)	$10.3 M ($7.3–$13.3 M)
Drug eruption	239 (194–283)	$6459 ($5326–$7592)	$1.5 M ($1.2–$1.9 M)
Congenital skin abnormalities	232 (183–280)	$45,521 ($26,889–$64,153)	$10.6 M ($5.5–$15.7 M)
Atopic dermatitis	225 (176–273)	$6818 ($5760–$7876)	$1.5 M ($1.1–$2.0 M)
Sweat gland disorders	176 (135–217)	$18,016 ($9809–$26,222)	$3.2 M ($1.6–$4.7 M)
Acne, rosacea, follicular disorders	62 (44–81)	$10,654 ($7614–$13,693)	$0.7 M ($0.4–$0.9 M)
Psoriasis	36 (21–50)	$15,382 ($6990–$23,775)	$0.5 M ($0.2–$0.9 M)
Hair and nail disorders	30 (16–44)	$14,599 ($6153–$23,045)	$0.4 M ($0.1–$0.8 M)
Bullous diseases	29 (13–45)	$22,721 ($7724–$37,718)	$0.7 M ($0.1–$1.2 M)
Pruritus	23 (11–35)	$6230 ($3809–$8652)	$0.1 M ($0.1–$0.2 M)
Nonmelanoma skin cancer	14 (4–23)	$20,367 ($9015–$31,720)	$0.3 M ($0.1–$0.5 M)
Melanoma	11 (8–21)	$28,643 ($18,474–$38,813)	$0.3 M ($0.0–$0.6 M)
Vitiligo, melasma	< 10	—	—

## 4 Discussion

This analysis of the 2022 KID reveals that the relative burden of pediatric dermatology admissions has declined substantially over the past decade, accounting for 2.3% of all pediatric hospitalizations compared to the 4.2% reported in 2012 [4]. The number of pediatric dermatology hospitalizations decreased from 74,229 in 2012 to 29,766 in 2022. This 59.9% decrease in dermatology‐specific hospitalizations was much steeper than the 25.7% decline seen in overall pediatric admissions (from approximately 1.77 million in 2012 to 1.31 million in 2022).

Several explanations are possible. Expanded access to outpatient therapies, greater availability of urgent dermatology consultation, and broader adoption of biologic and targeted agents may have shifted management of certain conditions away from inpatient care [11]. Shifts in where children seek acute dermatologic care also likely contribute. Many dermatologic complaints presenting to the emergency department are managed without admission and discharged after evaluation. In parallel, the expansion of urgent care centers over the past decade has created alternative sites for low‐acuity skin problems [12]. Together, these venue changes plausibly reduce inpatient dermatology admissions by resolving cases outside the hospital setting.

Despite the overall decrease in share, the demographic patterns observed in 2022 mirror many of those reported a decade earlier. Children from lower‐income communities and those insured by Medicaid were disproportionately represented. These findings are consistent with persistent disparities in access to timely outpatient dermatologic care, a theme also observed in other specialties where socioeconomic barriers may contribute to delayed presentation and higher hospitalization rates [12, 13, 14, 15]. The elevated odds of dermatologic hospitalization among Hispanic, Asian, and Native American children in 2022 warrant particular attention, as these groups were not significantly overrepresented in the 2012 analysis. Over the last decade, the U.S. child population has seen a significant increase in racial and ethnic diversity. Children are now more diverse than adults, with over half of all children under the age of five identifying as children of color in 2020. This demographic shift may also be influencing changes in the case mix across different racial and ethnic groups [16].

Financially, dermatology admissions in 2022 carried lower median costs and shorter lengths of stay than other pediatric admissions, consistent with the prior report [4]. Since 2012, the financial burden per case significantly increased, with the mean cost per admission rising from $5211 in 2012 (inflation‐adjusted to approximately $7025 in 2022) to $15,093 in 2022. This likely reflects both general increases in healthcare costs and the introduction of high‐cost therapeutics [17, 18]. Additionally, the large gap between our 2022 median cost ($6728) and this mean cost ($15,093) illustrates that the average is heavily skewed by high‐cost conditions such as cutaneous lymphomas and congenital skin abnormalities. These continue to generate disproportionately high costs per admission, underscoring the importance of early diagnosis, access to subspecialty care, and targeted outpatient management strategies [19, 20, 21].

An increase in the in‐hospital mortality rate from 0.03% in 2012 to 0.1% in 2022 was observed, which occurred even as total admissions fell significantly. The 2012 study reported that mortality was primarily driven by congenital skin abnormalities, including conditions such as epidermolysis bullosa and congenital ichthyoses. The 2022 data show a continued, though broader, concentration of deaths in high‐acuity conditions (including cutaneous lymphoma, viral diseases, and congenital skin abnormalities), whereas bacterial infections, the most common admission category, accounted for zero deaths. This mortality data supports that there may be a shift towards a more complex inpatient cohort.

The observed decline in dermatology admissions and the changes in mortality rates occurred against the backdrop of the U.S. transition from ICD‐9‐CM to ICD‐10‐CM in 2015, which expanded diagnostic specificity and may partly account for differences in categorization across years [5, 6]. While this increased granularity improves accuracy, it also complicates direct comparisons with earlier datasets. Nevertheless, the broader patterns of socioeconomic and regional disparities appear stable across coding systems.

This study has limitations inherent to administrative data. Findings rely on administrative coding and may be affected by misclassification. For example, ICD‐10 codes C82–C86 can include systemic lymphomas with skin involvement, which may overstate the burden attributed to primary cutaneous lymphoma. The use of the primary diagnosis field excludes admissions where skin disease was a secondary but clinically significant factor. Additionally, the database does not capture outpatient dermatology utilization, biologic therapy use, or teledermatology access, which may influence inpatient burden. Finally, although the study reports national estimates, the cross‐sectional design precludes assessment of longitudinal outcomes for individual patients. It is also essential to consider the impact of the COVID‐19 pandemic on our 2022 findings. The pandemic altered healthcare‐seeking behaviors, hospital capacity, and non‐urgent admission patterns. It is possible that the observed decrease in dermatology admissions was related to pandemic‐driven healthcare avoidance or shifts in hospital resources. While the data cannot quantify this effect, it represents a significant contextual difference from the 2012 data.

In summary, the national burden of inpatient pediatric dermatology has decreased over the past decade, yet disparities by socioeconomic status, payer, and region remain. Interventions to expand outpatient access, particularly for underserved populations, are likely to reduce preventable hospitalizations and address persistent inequities in care.

## Funding

The authors have nothing to report.

## Conflicts of Interest

P.A.L. reports research grants/funding from AbbVie, AOBiome, National Eczema Association; is on the speaker's bureau for AbbVie, Arcutis, Eli Lilly, Galderma, Hyphens Pharma, Incyte, La Roche‐Posay/L'Oreal, MyOR Diagnostics, Pfizer, Pierre‐Fabre Dermatologie, Regeneron/Sanofi Genzyme, Verrica; reports consulting/advisory boards for Alphyn, AbbVie, Almirall, Amyris, Arcutis, ASLAN, Boston Skin Science, Bristol‐Myers Squibb, Burt's Bees, Castle Biosciences, Codex Labs, Concerto Biosci, Dermavant, Eli Lilly, Galderma, Janssen, Johnson & Johnson, Kimberly‐Clark, LEO Pharma, Lipidor, L'Oreal, Merck, Micreos, MyOR Diagnostics, Regeneron/Sanofi Genzyme, Skinfix, Theraplex, UCB, Unilever, Verrica Yobee Care; stock options with Codex, Concerto Biosciences and Yobee Care. In addition, Dr. Lio has a patent pending for a Theraplex product with royalties paid and is a Board member and Scientific Advisory Committee Member of the National Eczema Association. The other authors report no conflicts of interest.

## Supporting information


**Table S1:** ICD‐10‐CM code prefixes for dermatology disease classification.

## Data Availability

The data that support the findings of this study are available from Healthcare Cost and Utilization Project (HCUP). Restrictions apply to the availability of these data, which were used under license for this study. Data are available from the author(s) with the permission of Healthcare Cost and Utilization Project (HCUP).

## References

[1] O. Burke , M. Hartoyo , R. Lin , R. S. Kirsner , and S. A. Elman , “The Financial Impact and Utilization of Inpatient Dermatology Services: Historical Insights and Future Implications,” Archives of Dermatological Research 317, no. 1 (2025): 374, 10.1007/s00403-025-03867-y. 39921720 PMC11807067

[2] CMS , “NHE Fact Sheet,” accessed August 26, 2025, https://www.cms.gov/data‐research/statistics‐trends‐and‐reports/national‐health‐expenditure‐data/nhe‐fact‐sheet.

[3] E. K. Collier , J. J. Yang , S. Sangar , S. T. Chen , J. T. Huang , and D. Q. Bach , “Evaluation of Emergency Department Utilization for Dermatologic Conditions in the Pediatric Population Within the United States From 2009‐2015,” Pediatric Dermatology 38, no. 2 (2021): 449–454, 10.1111/pde.14476. 33336810

[4] J. D. Arnold , S. Yoon , and A. Y. Kirkorian , “Inpatient Burden of Pediatric Dermatology in the United States,” Pediatric Dermatology 35, no. 5 (2018): 602–606, 10.1111/pde.13549. 29962044

[5] J. A. Hirsch , G. Nicola , G. McGinty , et al., “ICD‐10: History and Context,” American Journal of Neuroradiology 37, no. 4 (2016): 596–599, 10.3174/ajnr.A4696. 26822730 PMC7960170

[6] CMS , “Transitioning to ICD‐10,” accessed August 26, 2025, https://www.cms.gov/newsroom/fact‐sheets/transitioning‐icd‐10.

[7] C. M. Lombardi , L. R. Bullinger , and M. Gopalan , “Better Late Than Never: Effects of Late ACA Medicaid Expansions for Parents on Family Health‐Related Financial Well‐Being,” Inquiry: Journal of Health Care Organization, Provision, and Financing 59 (2022): 469580221133215, 10.1177/00469580221133215. PMC9661594 36354062

[8] E. Paskett , B. Thompson , A. S. Ammerman , A. N. Ortega , J. Marsteller , and D. Richardson , “Multilevel Interventions to Address Health Disparities Show Promise in Improving Population Health,” Health Affairs 35, no. 8 (2016): 1429–1434, 10.1377/hlthaff.2015.1360. 27503968 PMC5553289

[9] S. J. Willems , M. C. Castells , and A. P. Baptist , “The Magnification of Health Disparities During the COVID‐19 Pandemic,” Journal of Allergy and Clinical Immunology: In Practice 10, no. 4 (2022): 903–908, 10.1016/j.jaip.2022.01.032. 35131511 PMC8813673

[10] H. W. Lim , S. A. B. Collins , J. S. Resneck , et al., “The Burden of Skin Disease in the United States,” Journal of the American Academy of Dermatology 76, no. 5 (2017): 958–972.e2, 10.1016/j.jaad.2016.12.043. 28259441

[11] R. J. B. Driessen , L. A. Bisschops , E. M. M. Adang , A. W. Evers , P. C. M. Van De Kerkhof , and E. M. G. J. De Jong , “The Economic Impact of High‐Need Psoriasis in Daily Clinical Practice Before and After the Introduction of Biologics,” British Journal of Dermatology 162, no. 6 (2010): 1324–1329, 10.1111/j.1365-2133.2010.09693.x. 20163420

[12] A. R. Kreider , B. French , J. Aysola , B. Saloner , K. G. Noonan , and D. M. Rubin , “Quality of Health Insurance Coverage and Access to Care for Children in Low‐Income Families,” JAMA Pediatrics 170, no. 1 (2016): 43–51, 10.1001/jamapediatrics.2015.3028. 26569497 PMC8011294

[13] A. S. Algarni , S. M. Alshiakh , S. M. Alghamdi , et al., “From Rash Decisions to Critical Conditions: A Systematic Review of Dermatological Presentations in Emergency Departments,” Diagnostics 15, no. 5 (2025): 614, 10.3390/diagnostics15050614. 40075860 PMC11898758

[14] A. Creadore , S. Desai , S. J. Li , et al., “Insurance Acceptance, Appointment Wait Time, and Dermatologist Access Across Practice Types in the US,” JAMA Dermatology 157, no. 2 (2021): 181, 10.1001/jamadermatol.2020.5173. 33439219 PMC7807384

[15] S. B. Chaudhry , E. S. Armbrecht , Y. Shin , et al., “Pediatric Access to Dermatologists: Medicaid Versus Private Insurance,” Journal of the American Academy of Dermatology 68, no. 5 (2013): 738–748, 10.1016/j.jaad.2012.10.034. 23474423

[16] Bureau UC , Exploring the Racial and Ethnic Diversity of Various Age Groups (Census.gov), accessed November 6, 2025, https://www.census.gov/newsroom/blogs/random‐samplings/2023/09/exploring‐diversity.html.

[17] R. J. Willer , E. R. Coon , W. N. Harrison , and S. L. Ralston , “Trends in Hospital Costs and Levels of Services Provided for Children With Bronchiolitis Treated in Children's Hospitals,” JAMA Network Open 4, no. 10 (2021): e2129920, 10.1001/jamanetworkopen.2021.29920. 34698848 PMC8548950

[18] B. N. Rome , A. C. Egilman , and A. S. Kesselheim , “Trends in Prescription Drug Launch Prices, 2008‐2021,” JAMA 327, no. 21 (2022): 2145–2147, 10.1001/jama.2022.5542. 35670795 PMC9175077

[19] J. H. Choe , T. Yu , J. S. Abramson , and M. Abou‐El‐Enein , “Cost‐Effectiveness of Second‐Line Lisocabtagene Maraleucel in Relapsed or Refractory Diffuse Large B‐Cell Lymphoma,” Blood Advances 8, no. 2 (2024): 484–496, 10.1182/bloodadvances.2023011793. 38153350 PMC10837180

[20] J. J. Scarisbrick , M. Bagot , and P. L. Ortiz‐Romero , “The Changing Therapeutic Landscape, Burden of Disease, and Unmet Needs in Patients With Cutaneous T‐Cell Lymphoma,” British Journal of Haematology 192, no. 4 (2021): 683–696, 10.1111/bjh.17117. 33095448 PMC7894136

[21] A. Howles and J. Scarisbrick , “Cutaneous T‐Cell Lymphoma: Practical Recommendations to Enhance Clinical Practice,” British Journal of Hospital Medicine 82, no. 3 (2021): 1–6, 10.12968/hmed.2021.0055. 33792384

