# Supplementary material for: The Inpatient Burden of Pediatric Dermatology in the United States: A National Analysis of the 2022 Kids' Inpatient Database
Source: Pediatr Dermatol. 2026 Feb 13;43(3):615–21. doi: 10.1111/pde.70151 (PMC13206354; doi:10.1111/pde.70151)
Supplement: Supplementary file 1 — Table S1: ICD‐10‐CM code prefixes for dermatology disease classification. [file PDE-43-615-s001.docx]

**Supplemental Table 1.** ICD-10-CM Code Prefixes for Dermatology Disease Classification

| **Disease Category** | **ICD-10-CM Code Prefixes** |
| --- | --- |
| **Infections/Infestations** |  |
| Bacterial infections/infestations | A46, L01, L02, L03, L04, L05, L08, B85, B86, B87, B88 |
| Viral diseases | B00, B01, B02, B05, B06, B07, B08, B09 |
| Fungal infections | B35, B36, B37, B49 |
| **Inflammatory Disorders** |  |
| Connective tissue disorders | L92, L93, L94, M32, M33, M34, M35 |
| Atopic dermatitis | L20 |
| Other Eczema/Contact dermatitis | L21, L23, L24, L25, L30 |
| Urticaria | L50 |
| Psoriasis | L40 |
| Drug eruption | L270, L271 |
| Bullous diseases | L10, L11, L12, L13, L14 |
| **Growths/Neoplasms** |  |
| Noncancerous skin growths | D180, D22, D23, I781, Q825 |
| Cutaneous lymphoma | C82, C83, C84, C85, C86 |
| Melanoma | C43 |
| Nonmelanoma skin cancer | C44 |
| **Other Disorders** |  |
| Ulcers | I830, I832, L89, L97, L984 |
| Congenital skin abnormalities | Q80, Q81, Q82, Q84 |
| Sweat gland disorders | L73, L74, L75 |
| Hair and nail disorders | L60, L62, L63, L64, L65, L66, L67, L68 |
| Vitiligo, melasma | L80, L811 |
| Pruritus | L29 |
| Acne, rosacea, follicular disorders | L70, L71, L72 |
